# Characterization of Conserved MicroRNAs in Avocado Seed Weevils (Coleoptera: Curculionidae)

**DOI:** 10.3390/insects17101026

**Published:** 2026-10-06

**Authors:** Mariana Lizbeth Jiménez-Martínez, Eugenia Elizondo-González, Reyna Vargas-Abasolo, Iram Pablo Rodríguez-Sánchez, Gerardo de Jesús Trujillo-Rodríguez, María de Lourdes Ramírez-Ahuja

**Affiliations:** 1 Laboratorio de Fisiología Molecular y Estructural, Facultad de Ciencias Biológicas, Universidad Autónoma de Nuevo León, Avenida Universidad S/N Ciudad Universitaria, San Nicolás de los Garza 66455, Nuevo León, Mexico; mariana.jimenez80@gmail.com (M.L.J.-M.); iramrodriguez@gmail.com (I.P.R.-S.); 2 Departamento de Ecología Evolutiva, Instituto de Ecología, Universidad Nacional Autónoma de Mexico, Circuito Exterior, Ciudad Universitaria, Coyoacán 04510, Ciudad de México, Mexico; reynavargas.a@gmail.com

**Keywords:** small RNA sequencing, miRNA repertoire, Curculionidae, sequence homology, post-transcriptional regulation, reference-based annotation

## Abstract

Avocado seed weevils are pests of quarantine importance that can limit avocado production and commercialization. However, the molecular regulatory mechanisms of these insects remain largely unexplored, and information on their microRNA (miRNA) repertoires is scarce. This study performed a reference-based, in silico characterization and comparative analysis of putative conserved miRNAs in three avocado seed weevils of agricultural importance: *Conotrachelus perseae*, *Conotrachelus aguacatae*, and *Heilipus lauri*. Total RNA was extracted from pooled adult specimens, and five small RNA libraries representing species–sex combinations were sequenced using Illumina technology. Putative miRNAs were predicted with miRDeep2 using the *Leptinotarsa decemlineata* genome, *Tribolium castaneum* mature and precursor miRNAs, and additional mature-miRNA references from five insect orders. After data curation, annotations with a miRDeep2 score ≥ 5 and ≥10 mature reads were retained. The final catalogue contained 31 mature miRNA annotations, of which 20 were detected in all libraries. Pairwise Jaccard indices ranged from 0.677 to 0.931. miR-276-5p had the highest compositional relative abundance in four libraries, whereas miR-8-5p predominated in female *C. aguacatae*. Because each species–sex combination was represented by one pooled library, the results do not demonstrate differential abundance or sex specificity. The identified miRNAs remain putative because their identity and function were not experimentally validated. These results provide an initial catalogue and candidates for future genomic, expression, and functional studies.

## Simple Summary

Avocado-associated weevils are economically important pests, but little is known about the molecules that regulate their genes. MicroRNAs are short RNA molecules that help control gene activity and participate in many biological processes. We analyzed sequencing data from adult females and males of *Conotrachelus perseae* and *Heilipus lauri*, as well as adult females of *Conotrachelus aguacatae*, to identify putative conserved microRNA annotations and compare their occurrence among these insects. We detected 31 putative conserved microRNAs, of which 20 were found in all five sample groups analyzed, indicating that the three species share a substantial set of these regulatory molecules. MicroRNA-276-5p was the most abundant in the female and male libraries of *H. lauri* and *C. perseae*, whereas microRNA-8-5p was predominant in female *C. aguacatae*. Because each species and sex was represented by a single pooled sample and identification was based on comparison with sequences reported in another beetle, these findings are descriptive and require further confirmation. Nevertheless, this study provides an initial molecular resource for these avocado-associated weevils and identifies candidate microRNAs for future research on their biology and the development of more targeted pest-management strategies.

## 1. Introduction

The Curculionidae family is the largest within the order Coleoptera, comprising approximately 4600 genera and 51,000 described species [1]. Most curculionids are phytophagous, and many species are considered important pests of agricultural crops [2].

*Conotrachelus perseae* Barber and *Conotrachelus aguacatae* Barber, commonly known as small avocado seed weevils, and *Heilipus lauri* Boheman, known as the large avocado seed weevil, cause damage to avocado fruits [3,4]. These species are considered pests of quarantine importance and represent a constraint on avocado production and commercialization [5,6].

Research on avocado weevils has primarily focused on their morphology, taxonomy, distribution, and ecology [2,3,7]. However, their molecular regulatory mechanisms remain largely unexplored. To the best of our knowledge, no previous studies have examined the microRNA repertoire of these avocado seed weevils.

MicroRNAs (miRNAs) are small non-coding RNAs involved in the post-transcriptional regulation of gene expression [8]. Canonical miRNA biogenesis in insects begins with the transcription of miRNA loci by RNA polymerase II, generating primary miRNA (pri-miRNA) transcripts. These transcripts are processed in the nucleus by the Drosha–Pasha complex, which excises a precursor miRNA (pre-miRNA) hairpin of approximately 70 nucleotides. Following Exportin-5-mediated transport to the cytoplasm, Dicer-1 cleaves the pre-miRNA to generate a ~22-nucleotide miRNA duplex. The mature miRNA strand is subsequently loaded into an Argonaute-1 (Ago1)-containing RNA-induced silencing complex (RISC), which mediates post-transcriptional gene regulation by targeting complementary sequences, typically within the 3′ untranslated regions of mRNAs, leading primarily to translational repression and mRNA destabilization or degradation [9].

In insects, miRNA-mediated regulation represents one of the major small-RNA pathways, together with the siRNA and piRNA pathways [10]. Importantly, the endogenous miRNA pathway differs from the dsRNA-triggered siRNA pathway commonly exploited for RNAi-based pest control, in which long double-stranded RNA is processed primarily by Dicer-2 into siRNAs that guide Ago2-containing RISC complexes toward highly complementary target transcripts [10,11].

In insects, miRNAs have been associated with several biological processes, including sexual differentiation, oogenesis, embryogenesis, metamorphosis, and insecticide resistance [9,12]. Their regulatory roles have also led to increasing interest in miRNAs as potential molecular targets for pest management. Alterations in miRNA expression may affect insect development, survival, and responses to insecticides [8]. Nevertheless, the study of miRNAs in agriculturally important weevils remains limited.

Therefore, this study aimed to generate a reference-based catalogue of putative miRNAs in *C. perseae*, *C. aguacatae*, and *H. lauri*. Specifically, we examined annotation overlaps among the available sequencing libraries, descriptive relative-abundance patterns, and cross-reference support using miRNA sequences reported from other insect groups. The resulting data provide an initial molecular resource for these avocado seed weevils and a basis for future expression and experimental validation studies.

## 2. Materials and Methods

### 2.1. Biological Material

Adult specimens of *Conotrachelus perseae* and *Heilipus lauri* were collected in September 2018 from infested fruits of *Persea americana* var. Hass and var. mexicana in Meyuca de Morelos, Coatepec de Harinas, Estado de México, Mexico (18°86′43″ N, 99°78′66″ W). Adult specimens of *Conotrachelus aguacatae* were collected in June 2018 from infested avocado fruits in Comonfort, Guanajuato, Mexico (20°43′50.4″ N, 100°47′40.8″ W).

The biological material consisted exclusively of adult specimens. For *H. lauri* and *C. perseae*, 15 males and 15 females were analyzed per species. Individuals were separated by sex and processed as independent pooled samples, resulting in one male pool and one female pool for each species. For *C. aguacatae*, only females were available; therefore, 15 adult females were processed as a single pooled sample. In total, five pooled samples were analyzed: male and female pools of *H. lauri*, male and female pools of *C. perseae*, and one female pool of *C. aguacatae*.

### 2.2. RNA Extraction and Small RNA Sequencing

Total RNA was extracted from each pooled sample using TRIzol reagent according to the manufacturer’s instructions (Invitrogen/Thermo Fisher Scientific, Carlsbad, CA, USA). RNA concentration and purity were assessed by spectrophotometry, whereas RNA integrity was evaluated by agarose gel electrophoresis.

RNA samples meeting the required quality and quantity criteria were sent to BGI Global Genomics Services (Yantian District, Shenzhen, China). Small RNA enrichment, library preparation, adapter removal, and sequencing were performed by BGI according to its standard protocols. No additional trimming or quality filtering was performed by the authors after receiving the processed sequencing data. Small RNA libraries were sequenced using Illumina Solexa technology, generating single-end reads ranging from 18 to 45 nucleotides in length.

Quality-control metrics for the processed FASTQ files were assessed using FastQC v0.11.9 and are provided in the supplementary dataset. These metrics include the total number of sequences in each processed FASTQ file, sequence-length range, GC content, and sequences flagged as poor quality.

### 2.3. In Silico Prediction of Putative miRNAs

Putative miRNAs were predicted using miRDeep2 [13]. Because reference genomes were not available for the three avocado weevil species, the genome of *Leptinotarsa decemlineata* (Coleoptera: Chrysomelidae) (NCBI RefSeq assembly GCF_025532065.1) was used as a heterologous genomic reference. Mature and precursor miRNA sequences reported for *Tribolium castaneum* in miRBase v22 were used as secondary reference sequences [14]. *L. decemlineata* was selected as the heterologous genomic reference because of its closer phylogenetic relationship to the avocado-associated weevils, whereas *T. castaneum* was retained as the source of known mature and precursor miRNA sequences available in miRBase.

Sequences shorter than 18 nucleotides were excluded from the analysis. A minimum miRDeep2 score of ≥5 was selected as a conservative operational cutoff, and its effect on catalogue size was evaluated through sensitivity analyses using score thresholds of ≥4, ≥5, ≥6, and ≥10. Candidate sequences showing homology to previously reported miRNAs were initially classified as putatively conserved. Because miRNA prediction was based exclusively on computational analyses and no additional molecular or functional validation was performed, all sequences reported in this study were considered putative miRNAs.

### 2.4. Cross-Reference Annotation Support

To assess additional reference support for the retained miRNA annotations, each sequencing library was analyzed in five separate miRDeep2 runs. The genomic reference, *Tribolium castaneum* mature and precursor miRNA sequences, and all other analysis parameters were kept constant, whereas the mature_other reference set was changed among Diptera, Hemiptera, Hymenoptera, Lepidoptera, and Orthoptera.

For each retained annotation, support was summarized according to the insect-order reference sets in which a same-seed miRNA match was reported. These order-specific runs were not treated as independent observations or biological replicates. Rather, they were used as cross-reference annotation support. Accordingly, this analysis was not interpreted as an independent phylogenetic test of miRNA conservation.

### 2.5. Data Curation and Comparative Analysis of Putatively Conserved miRNAs

All downstream data processing and visualization were performed in R version 4.4.1 “Race for Your Life” [15]. Data were imported from an Excel database and organized using packages from the tidyverse ecosystem [16]. The comparative dataset comprised five sequencing libraries: female and male *C. perseae*, female *C. aguacatae*, and female and male *H. lauri*. Each library represented a single pool of 15 adult specimens from the corresponding species–sex combination. Because only one pooled sequencing library was available for each combination, all subsequent comparisons were considered descriptive.

#### 2.5.1. Data Curation

In the original dataset, each miRDeep2 locus was repeated for every insect order in which a homologous miRNA sequence was identified. Because these order-specific records did not represent independent observations, mature-read counts were not summed across rows.

The dataset was first collapsed by sequencing library and miRDeep2 locus. For each locus, the most frequent mature miRNA annotation and consensus sequence were retained, together with the maximum mature-read count. The resulting records were subsequently collapsed by sequencing library and mature miRNA annotation. When the same annotation was associated with multiple loci displaying identical or potentially redundant read assignments, the maximum count was retained rather than summing counts to minimize potential double counting. Records presenting inconsistent annotations, sequences, or counts were exported for manual quality-control inspection.

#### 2.5.2. Detection and Presence–Absence Analyses

A mature miRNA was considered detected in a sequencing library when at least 10 mature reads remained after data curation and collapsing. This cutoff was used as an operational detection threshold and not as a criterion of statistical significance. To assess the sensitivity of the number of detected miRNAs to this decision, additional summaries were generated using thresholds of 1, 5, 10, and 20 mature reads.

Mature miRNAs meeting the 10-read threshold in at least one sequencing library were retained for the principal analyses. A binary presence–absence matrix was subsequently constructed for the five libraries.

Shared and library-specific mature miRNAs were visualized using an UpSet-style plot, which provides a matrix-based representation of intersections among multiple sets [17]. All 10 possible pairwise comparisons among the five libraries were also evaluated. For each comparison, the numbers of miRNAs shared between the two libraries, exclusive to each library, and present in the union of both libraries were calculated.

Similarity between pairs of libraries was quantified using the Jaccard index and the Sørensen–Dice coefficient, which describe the degree of overlap between two sets of detected miRNAs [18,19]. Pairwise comparisons were classified as female-male comparisons within the same species, comparisons among females of different species, comparisons among males of different species, or comparisons involving both different species and different sexes.

#### 2.5.3. Descriptive Abundance Analysis

For each sequencing library, mature-read counts were normalized relative to the total collapsed count of annotated miRNAs and expressed per million annotated miRNA reads. Mature miRNAs with fewer than 10 reads were assigned an abundance of zero in the principal analyses. Normalized values were transformed as log2(x + 1) for visualization. Pairwise log2 abundance ratios were calculated using a pseudocount of 0.5 to avoid undefined values when a miRNA was absent from one library. These ratios were interpreted descriptively and were not used for differential-expression inference.

### 2.6. Distribution Across Insect Orders

To examine the taxonomic distribution of the putatively conserved miRNAs, duplicate combinations of mature miRNA and insect order were removed. A binary presence–absence matrix was then generated for Diptera, Hemiptera, Hymenoptera, Lepidoptera, and Orthoptera.

The number and proportion of retained mature miRNAs represented in each insect order were calculated both globally and separately for each weevil sequencing library. These patterns were visualized using bar plots, a library-by-order dot plot, and a binary heatmap. In the dot plot, point size represented the number of retained mature miRNAs with a homologous sequence in the corresponding insect order, whereas point color represented the proportion of retained miRNAs from each library represented in that order.

### 2.7. Data Visualization and Statistical Limitations

Figures were generated using ggplot2 [16], The UpSet-style figure was assembled from intersection-size and presence–absence panels. Scientific names were abbreviated and italicized in the figures, and female and male libraries were identified using sex symbols. The prefix tca- was retained in the processed tables for traceability to the miRBase reference but omitted from graphical axis labels for readability. Because biological replication was unavailable within each species–sex combination, no differential-expression analyses, hypothesis tests, confidence intervals, or *p*-values were calculated. Differences in miRNA detection, pairwise similarity, and compositional relative abundance were therefore interpreted exclusively as descriptive patterns among the five available pooled sequencing libraries. Moreover, all miRNAs included in these analyses were considered putative because their identity and biological functions were not experimentally validated.

## 3. Results

### 3.1. Curated Catalogue of Putative Conserved miRNAs

The five order-specific runs generated for each library were consolidated before filtering to prevent repeated records from inflating miRNA counts or abundances. Mature-read counts remained consistent across the order-specific runs, although miRDeep2 scores varied according to the reference used. After applying a minimum miRDeep2 score of 5 and a minimum abundance of 10 mature reads, 31 unique putative mature miRNA annotations were retained across the five libraries.

The number of retained annotations ranged from 23 to 29 per library. Female and male *H. lauri* contained 23 and 26 annotations, respectively, whereas female and male *C. perseae* contained 27 and 29 annotations. The female *C. aguacatae* library contained 26 annotations (Figure 1). The corresponding numbers of retained predicted loci were 24, 27, 28, 30, and 27, respectively. In each library, the difference between the number of loci and mature miRNA annotations resulted from the detection of miR-2-3p at two predicted loci. The total mature reads assigned to the retained catalogue ranged from 10,676 in female *H. lauri* to 142,760 in male *C. perseae*.

### 3.2. Shared and Library-Associated miRNA Repertoires

Of the 31 mature miRNA annotations detected across the complete dataset, 20 were present in all five libraries, representing 64.5% of the combined catalogue (Figure 2). This shared group included miR-276-5p, miR-8-5p, miR-34-5p, miR-2-3p, miR-281-5p, miR-71-5p, miR-31-5p, miR-317-3p, miR-184-3p, and other conserved annotations.

Three annotations, miR-210-5p, miR-282-5p, and miR-932-5p, were detected in all libraries except female *H. lauri*. Bantam-5p and miR-2944c-3p were shared by the three *Conotrachelus* libraries but were not detected in either *H. lauri* library. Only two annotations occurred in a single library: miR-305-5p in female *H. lauri* and miR-965-3p in male *C. perseae*. Given the absence of biological replicates, these detections are considered library-associated rather than species- or sex-specific.

### 3.3. Similarity in miRNA Presence–Absence Profiles

Pairwise Jaccard indices ranged from 0.677 to 0.931, indicating substantial overlap among the detected miRNA repertoires (Figure 3). The greatest similarity occurred between female and male *C. perseae* (Jaccard = 0.931). These libraries shared 27 annotations, and the male library contained two additional annotations. Female and male *H. lauri* shared 21 annotations from a union of 28, resulting in a Jaccard index of 0.750.

The lowest similarity was observed between female *H. lauri* and male *C. perseae* (Jaccard = 0.677), which shared 21 of the 31 annotations detected in either library. The female *C. aguacatae* library showed its greatest similarity to female *C. perseae* (Jaccard = 0.893; 25 shared annotations). These indices describe similarity in detection patterns and do not incorporate differences in read abundance.

### 3.4. Relative Abundance Patterns

The retained miRNA catalogue showed a concentrated abundance distribution in all five libraries. The two most abundant annotations in each library accounted for between 46.30% and 53.16% of the corresponding retained miRNA reads.

MiR-276-5p was the most abundant annotation in four libraries, representing 31.80% of retained miRNA reads in female *H. lauri*, 27.27% in male *H. lauri*, 27.46% in female *C. perseae*, and 26.98% in male *C. perseae*. In female *C. aguacatae*, miR-8-5p was the most abundant annotation at 32.32%, whereas miR-276-5p ranked second at 19.71% (Figure 4).

MiR-8-5p also represented 21.36% of the female *H. lauri* catalogue and 23.88% of the male *C. perseae* catalogue. Its relative abundance was lower in male *H. lauri* and female *C. perseae*, at 10.55% and 12.55%, respectively. MiR-34-5p was the second most abundant annotation in male *H. lauri* and female *C. perseae*, representing 19.04% and 20.27% of their retained reads. Other recurrent high-abundance annotations included miR-2-3p, miR-281-5p, miR-71-5p, miR-31-5p, and miR-317-3p. These values represent within-library relative abundances and were used only for descriptive comparisons (Figure 5).

### 3.5. Sensitivity to Filtering Thresholds and Support from Other Insect Orders

The number of retained annotations was strongly influenced by the minimum miRDeep2 score. At a score threshold of 4, the libraries retained between 33 and 50 annotations. Applying the selected threshold of 5 reduced this range to 23–29 annotations. At thresholds of 6 and 10, only 9–12 annotations were retained per library.

In contrast, the results were comparatively stable across mature-read thresholds. Thresholds of 5 and 10 reads produced identical catalogue sizes in all libraries. Reducing the threshold to one read added no annotations to either *H. lauri* library and between one and two annotations to each *Conotrachelus* library. Increasing the threshold to 20 reads retained 21 annotations in female *H. lauri*, 25 in male *H. lauri*, 26 in female *C. perseae*, 27 in male *C. perseae*, and 22 in female *C. aguacatae*.

Most retained annotations had a same-seed match in at least three of the five insect-order reference sets. This included 21 of 23 annotations in female *H. lauri*, 25 of 26 in male *H. lauri*, 26 of 27 in female *C. perseae*, 28 of 29 in male *C. perseae*, and 25 of 26 in female *C. aguacatae*. Same-seed matches were most frequent in the Diptera, Lepidoptera, and Hymenoptera reference sets, followed by Hemiptera, whereas Orthoptera provided the fewest matches (Figure 6). These comparisons indicate cross-reference support for the retained annotations but do not constitute an independent evolutionary or phylogenetic analysis.

## 4. Discussion

The reference-based reanalysis identified 31 putative conserved mature miRNA annotations across the five pooled libraries, of which 20 were detected in all libraries. This shared set represented 64.5% of the complete catalogue and indicates substantial overlap in the detected miRNA repertoires of the three avocado-associated weevil species. The number of retained annotations varied moderately among libraries, from 23 in female *Heilipus lauri* to 29 in male *Conotrachelus perseae*. Pairwise Jaccard indices ranged from 0.677 to 0.931. The highest similarity was observed between the female and male *C. perseae* libraries, whereas the corresponding comparison between the two *H. lauri* libraries produced a lower value of 0.750. These indices describe similarity in miRNA presence–absence profiles and should not be interpreted as measures of abundance similarity or differential expression.

The intersection analysis showed that most retained miRNAs belonged to a broadly shared repertoire, whereas only a small number displayed restricted detection patterns. For example, bantam-5p and miR-2944c-3p were detected in the three *Conotrachelus* libraries, miR-305-5p was detected only in the female *H. lauri* library, and miR-965-3p was detected only in the male *C. perseae* library. These patterns identify candidates for future investigation but cannot establish species specificity, sex specificity, or true absence from the remaining groups. Detection may have been influenced by biological variation, tissue composition, sequencing depth, heterologous mapping, or the filtering thresholds. In addition, each species–sex combination was represented by a single pooled library, and *C. aguacatae* was represented only by females.

The sensitivity analysis demonstrated that the size of the retained catalogue was strongly affected by the miRDeep2 score cutoff. Depending on the library, 33–50 annotations were retained at a threshold of 4, compared with 23–29 at the selected threshold of 5 and only 9–12 at thresholds of 6 and 10. A cutoff of 5 therefore produced a more conservative catalogue than a cutoff of 4 without restricting the analysis to the substantially smaller group of candidates retained at higher thresholds. Nevertheless, the pronounced decrease between score thresholds emphasizes that the resulting catalogue depends on this operational decision and supports presenting the sequences as putative annotations. In contrast, the read-count sensitivity analysis produced identical catalogue sizes at thresholds of 5 and 10 reads, while using a one-read threshold added no more than two annotations per library. Thus, the principal results were comparatively stable around the selected minimum of 10 mature reads, although this cutoff represents an operational detection threshold and not a criterion of statistical significance.

The analysis combined the genome of *Leptinotarsa decemlineata* with mature and precursor miRNA sequences reported for *Tribolium castaneum*. The use of *T. castaneum* annotations provided a coleopteran reference repertoire containing both deeply conserved and lineage-associated insect miRNAs [20]. However, the use of heterologous genomic and miRNA references may have limited the recovery of divergent, lineage-specific, or species-specific miRNAs and may also have affected precursor mapping and annotation. Consequently, the resulting catalogue should be interpreted as a reference-supported subset of the miRNAs potentially present in the three weevil species rather than as a complete description of their miRNA repertoires.

Cross-reference comparisons provided additional support for most retained annotations. More than 91% of the retained miRNAs in each library were supported by mature-miRNA reference sets from at least three of the five insect orders examined. Support was most frequent with the Diptera, Lepidoptera, and Hymenoptera reference sets and least frequent with Orthoptera. These differences may reflect variation in database coverage, the number of represented species, sequence divergence, and the completeness of miRNA annotation among insect orders. The five order-specific analyses used the same sequencing library and genomic reference and differed only in the references sequences supplied to miRDeep2. They therefore represent cross-reference annotation support rather than independent biological replication or a formal phylogenetic test of miRNA conservation. Moreover, because the analytical strategy used previously reported miRNA sequences, it was better suited to recovering conserved annotations than to identifying lineage-specific or novel miRNAs. In the absence of species-specific genomic confirmation and experimental validation, the bona fide miRNA identity of these candidates cannot be definitively established.

The abundance profiles were dominated by a small group of annotations. miR-276-5p was the most abundant miRNA in the female and male libraries of both *H. lauri* and *C. perseae*, accounting for 26.98–31.80% of the retained annotated miRNA reads in these libraries. In female *C. aguacatae*, miR-8-5p was predominant at 32.32%, followed by miR-276-5p at 19.71%. Together, the two most abundant annotations represented approximately 46.3–53.2% of the retained reads in each library, indicating that the compositional profiles were concentrated in a small number of miRNAs.

Members of the miR-276 family participate in several regulatory processes in other insects. In *Drosophila melanogaster*, miR-276a and miR-276b have been associated with circadian and behavioral regulation [21,22]. In *Locusta migratoria*, miR-276 has also been linked to juvenile-hormone biosynthesis, developmental timing, and reproduction through regulation of juvenile hormone acid methyltransferase [20,23]. The consistent abundance of miR-276-5p in the weevil libraries makes it a candidate for subsequent investigation. However, sequence-based annotation does not establish equivalence with a particular *Drosophila* paralog, conservation of the reported targets, or an equivalent physiological function in these weevils.

The retained miR-8-5p annotation was detected in all five libraries and was especially abundant in female *C. aguacatae* and male *C. perseae*. Members of the miR-8 family regulate growth and developmental signaling in *D. melanogaster* [24,25], and experimental manipulation of miR-8-3p in *T. castaneum* has demonstrated roles in appendage development and metamorphosis [26]. Nevertheless, the annotation retained in the present study corresponds to the 5p arm. Consequently, the functions reported specifically for miR-8-3p cannot be directly assigned to miR-8-5p, and the observed abundance patterns do not demonstrate regulated arm selection.

miR-34-5p was also detected in all five libraries and was among the most abundant annotations, particularly in male *H. lauri* and female *C. perseae*. In adult *D. melanogaster*, miR-34 contributes to the maintenance of neural integrity during aging and modulates neurodegenerative processes [27]. Other annotations detected across all five libraries included miR-281-5p, miR-2-3p, and miR-71-5p. Homologous miRNAs participate in ecdysone signaling, wing development, Notch-associated regulation, and reproduction in other insect models [28,29,30]. These studies provide comparative biological context but do not demonstrate conservation of the same targets, expression sites, or biological functions in avocado-associated weevils.

The reported abundance values are compositional because each percentage was calculated relative to the total retained annotated miRNA reads within its corresponding library. A high percentage therefore indicates that an annotation constituted a large fraction of the retained reads in that library, but it is not an absolute measure of expression. Differences among libraries may also be influenced by changes in the abundance of other miRNAs. Without biological replication, these values cannot support differential-expression testing, confidence intervals, or conclusions about sex-associated regulation. The use of complete adult specimens also prevents determination of the tissues responsible for the observed abundance patterns.

Future studies should incorporate independent biological replicates for each species and sex, including a male *C. aguacatae* group, together with tissue-specific or dissected-organ sequencing. Species-specific genome assemblies and experimentally supported precursor annotations would reduce dependence on heterologous references. Integration of small-RNA and messenger-RNA profiles, followed by target prediction using species-specific 3′ untranslated regions, could identify candidate regulatory relationships. Selected miRNAs, particularly miR-276-5p, miR-8-5p, miR-34-5p, miR-281-5p, miR-2-3p, and miR-71-5p, should be prioritized for future experimental validation using RT-qPCR, reporter assays, and RNAi-based or other gain- or loss-of-function approaches. Until such validation is performed, the present dataset should be regarded as a descriptive catalogue of putative conserved miRNAs and as a source of experimentally testable hypotheses in these three avocado-associated weevils.

## 5. Conclusions

This study provides an updated reference-based catalogue of putative conserved miRNAs in *Conotrachelus perseae*, *Conotrachelus aguacatae*, and *Heilipus lauri*. After data curation and application of a miRDeep2 score threshold of ≥5 and a minimum detection threshold of 10 mature reads, 31 mature miRNA annotations were retained, of which 20 were detected in all five libraries. Pairwise Jaccard indices ranged from 0.677 to 0.931, indicating substantial overlap in the detected miRNA repertoires. miR-276-5p was the most abundant annotation in the female and male libraries of *H. lauri* and *C. perseae*, whereas miR-8-5p was predominant in female *C. aguacatae*. Most retained annotations also received support from multiple insect-order reference sets, although this evidence represents cross-reference annotation support rather than independent confirmation of evolutionary conservation.

The results are descriptive because each species–sex combination was represented by a single pooled library, *C. aguacatae* was represented only by females, whole adult specimens were analyzed, and miRNA identification relied on heterologous genomic and miRNA references. Consequently, the observed patterns do not demonstrate differential expression, sex specificity, species specificity, or functional equivalence with homologous miRNAs characterized in other insects. Despite these limitations, the dataset expands the molecular resources available for avocado-associated weevils and identifies candidate miRNAs for future studies incorporating biological replication, species-specific genomic resources, tissue-level analyses, target identification, and experimental validation.

## Figures and Tables

**Figure 1:**
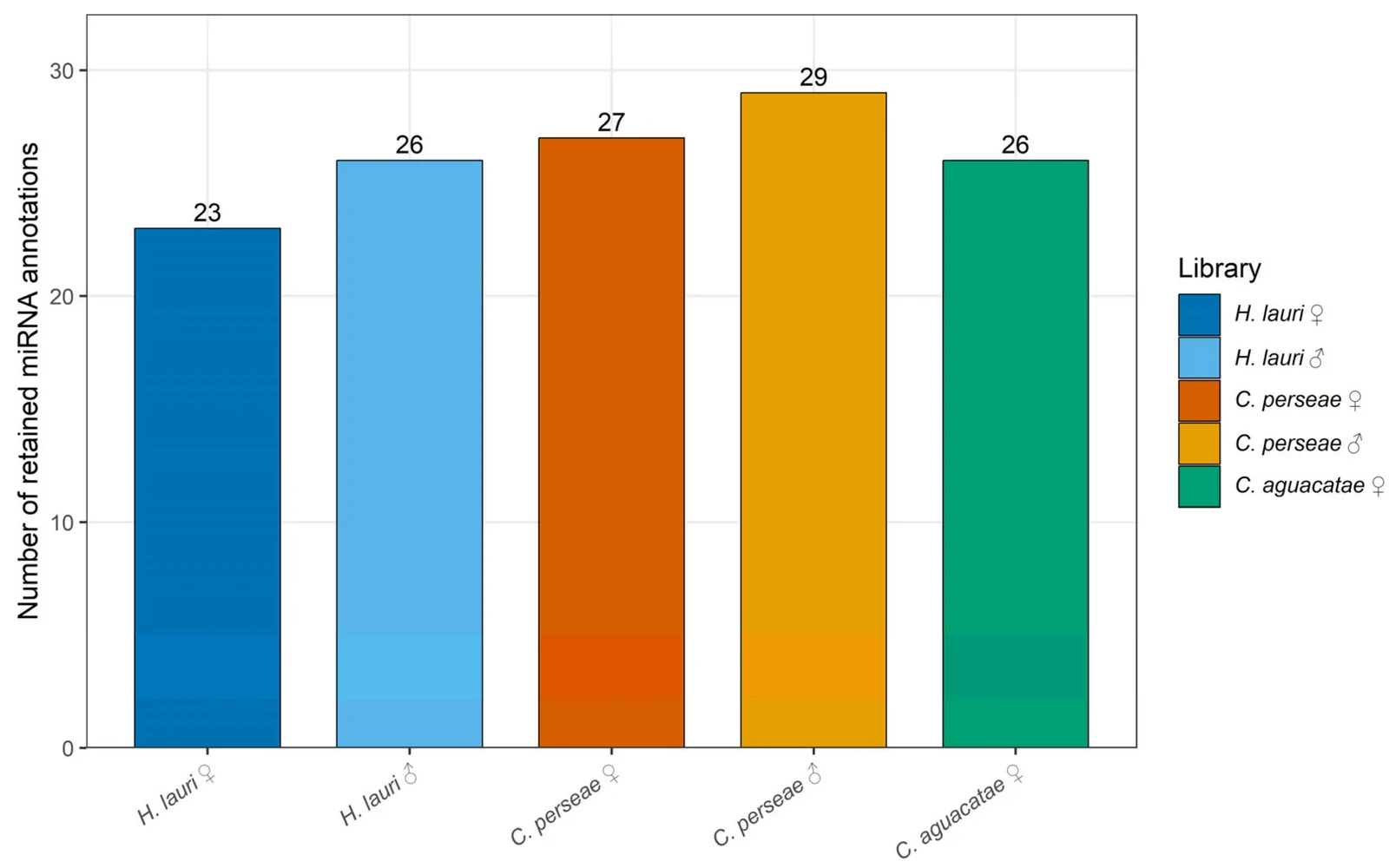
Number of retained mature miRNA annotations in each sequencing library. Bars show the number of mature miRNA annotations retained after data curation and application of a maximum miRDeep2 score threshold of ≥5 and a minimum detection threshold of 10 mature reads. Each library represents one pooled sample of 15 adult specimens. Values above the bars indicate the number of retained annotations. ♀, female; ♂, male.

**Figure 2:**
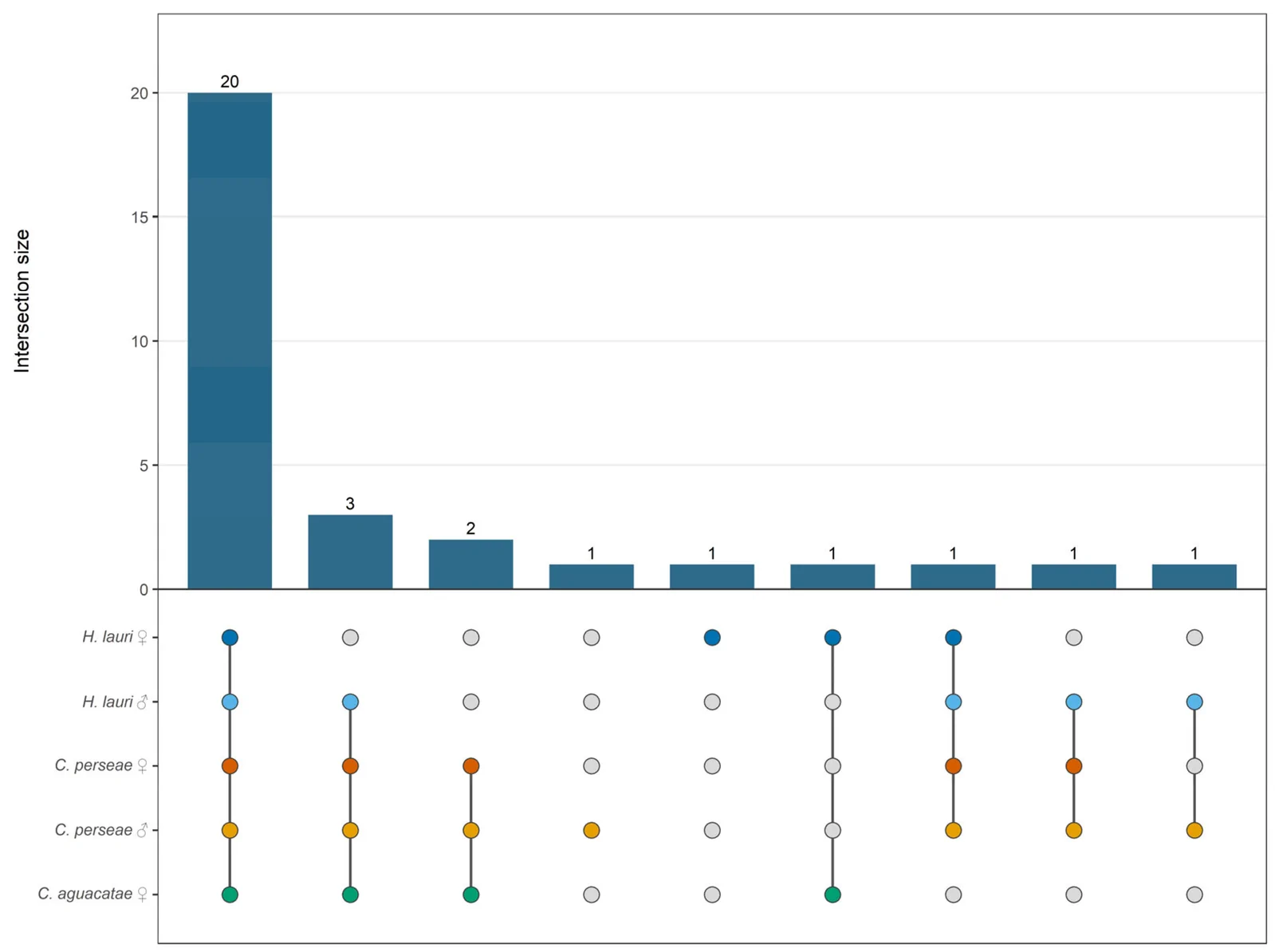
Intersection patterns of retained mature miRNAs among the five sequencing libraries. The UpSet plot summarizes the presence–absence patterns of the 31 mature miRNA annotations retained in at least one library. Upper bars and their labels indicate the number of miRNAs in each exact intersection. In the lower matrix, colored points identify the libraries included in each intersection, gray points indicate libraries not included, and connecting lines link libraries belonging to the same intersection. The largest intersection comprises 20 mature miRNAs detected in all five libraries. ♀, female; ♂, male.

**Figure 3:**
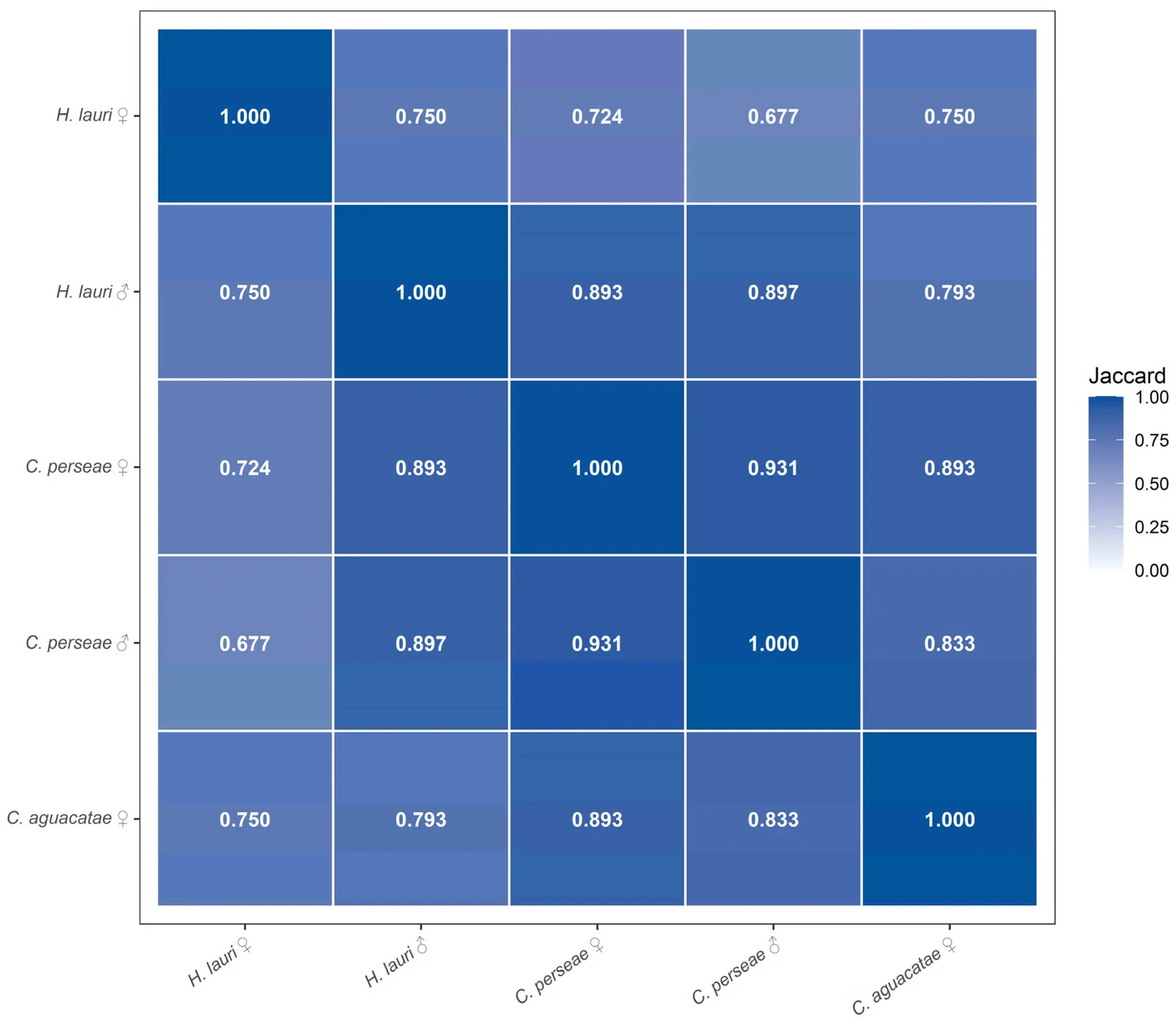
Pairwise similarity of mature miRNA repertoires among the five sequencing libraries. Heatmap showing Jaccard similarity indices calculated from the presence–absence profiles of mature miRNAs detected with ≥10 reads. Values range from 0 to 1, with values closer to 1 indicating greater overlap between the miRNA repertoires of two libraries. The diagonal represents comparisons of each library with itself. Numerical values are displayed within the corresponding cells. ♀, female; ♂, male.

**Figure 4:**
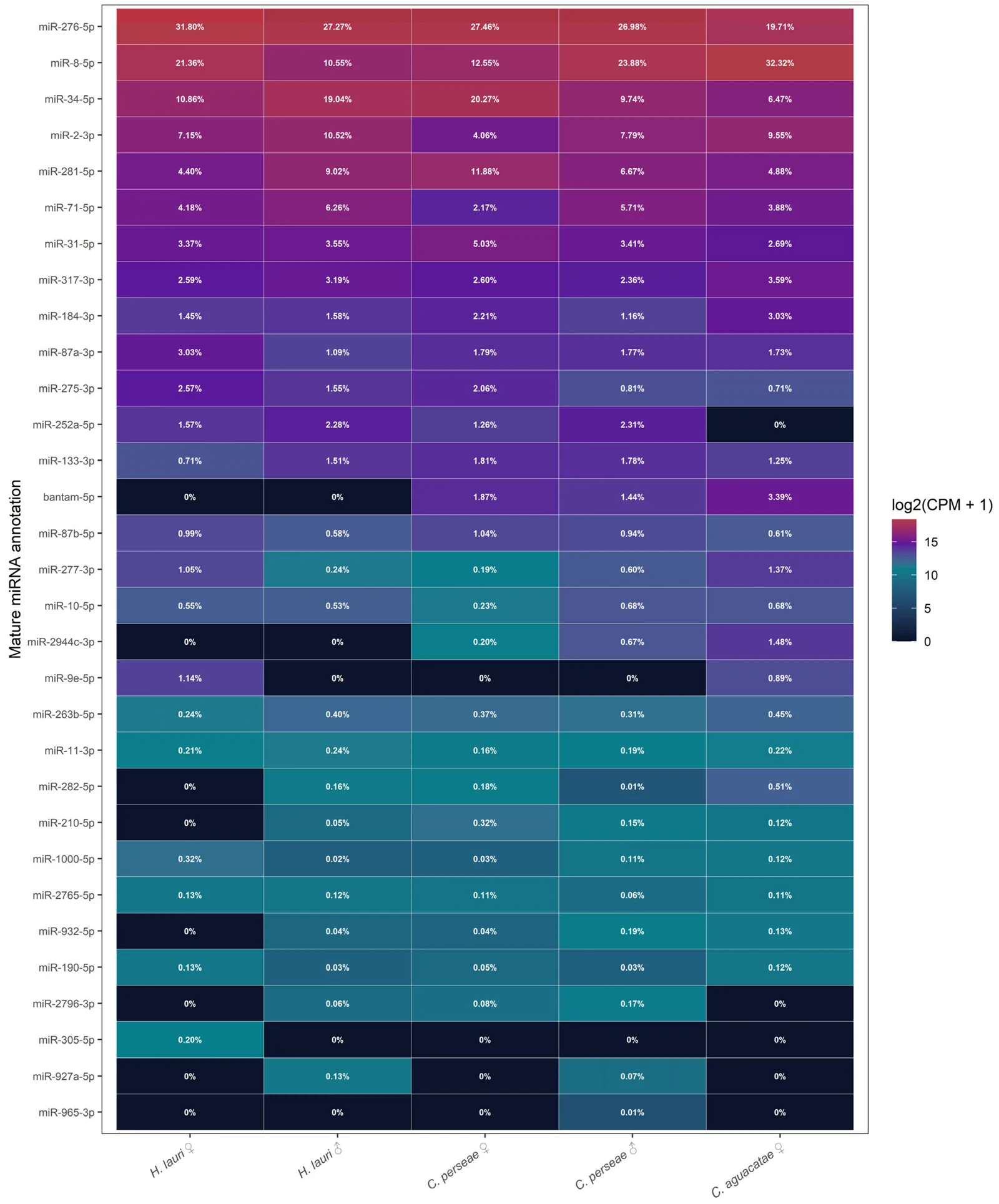
Relative abundance profiles of retained mature miRNAs across the five sequencing libraries. The heatmap includes all 31 mature miRNA annotations retained in at least one library and orders them according to their mean abundance across libraries. Cell color represents normalized abundance expressed as log2(CPM + 1), where CPM denotes counts per million retained annotated miRNA reads. Values printed within the cells represent the percentage of the total retained mature-miRNA reads in each library. Annotations with fewer than 10 mature reads in a library were assigned an abundance of zero. ♀, female; ♂, male.

**Figure 5:**
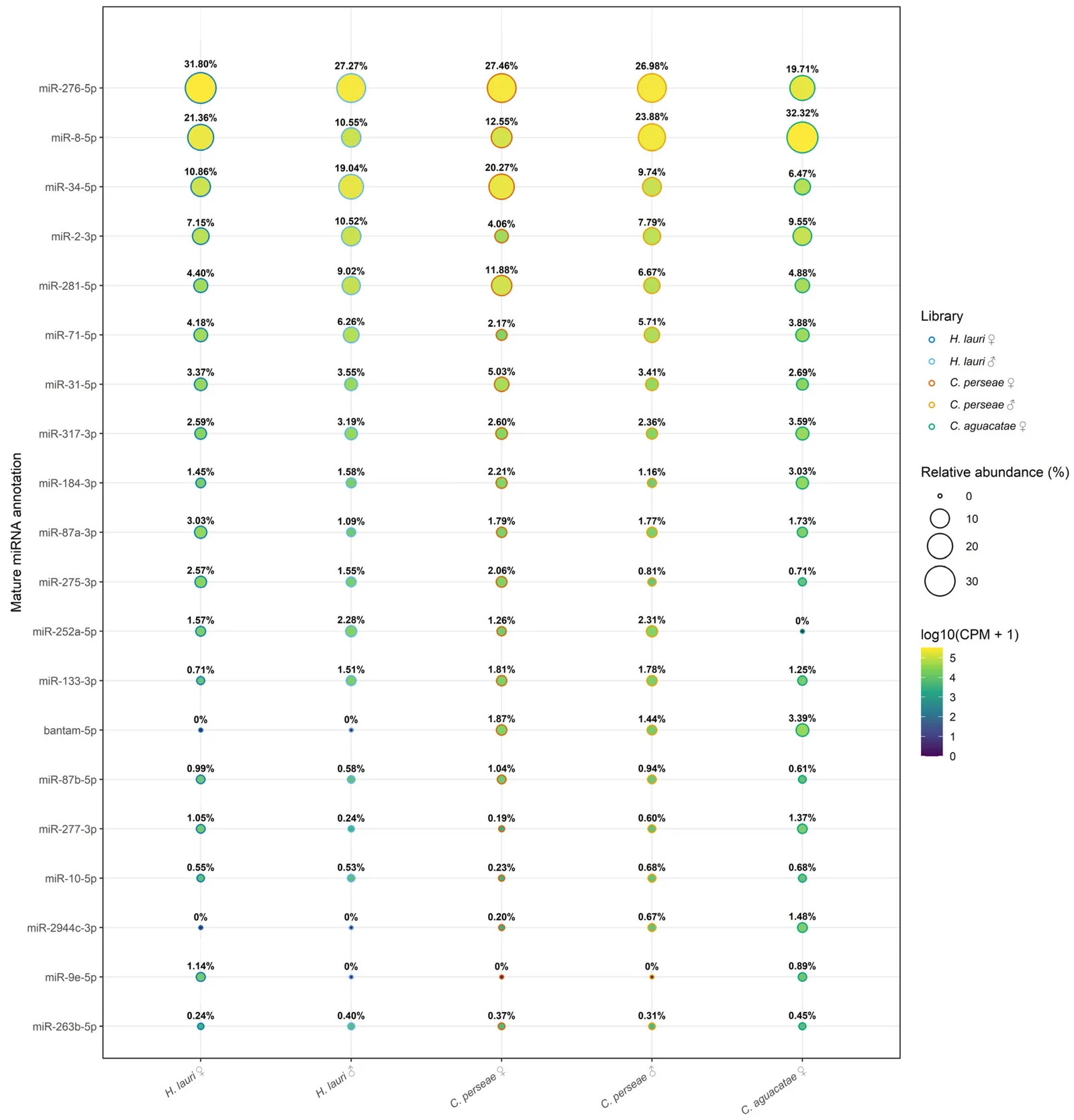
Relative abundance of the 20 most abundant mature miRNAs across the five sequencing libraries. The plot includes the 20 mature miRNA annotations with the highest mean relative abundance across all libraries. Point size represents relative abundance as a percentage of the total retained mature-miRNA reads within each library, whereas point fill represents log10(CPM + 1). Point-outline colors identify the corresponding sequencing libraries. Percentages are displayed above each point. Annotations with fewer than 10 mature reads in a library were assigned an abundance of zero. ♀, female; ♂, male.

**Figure 6:**
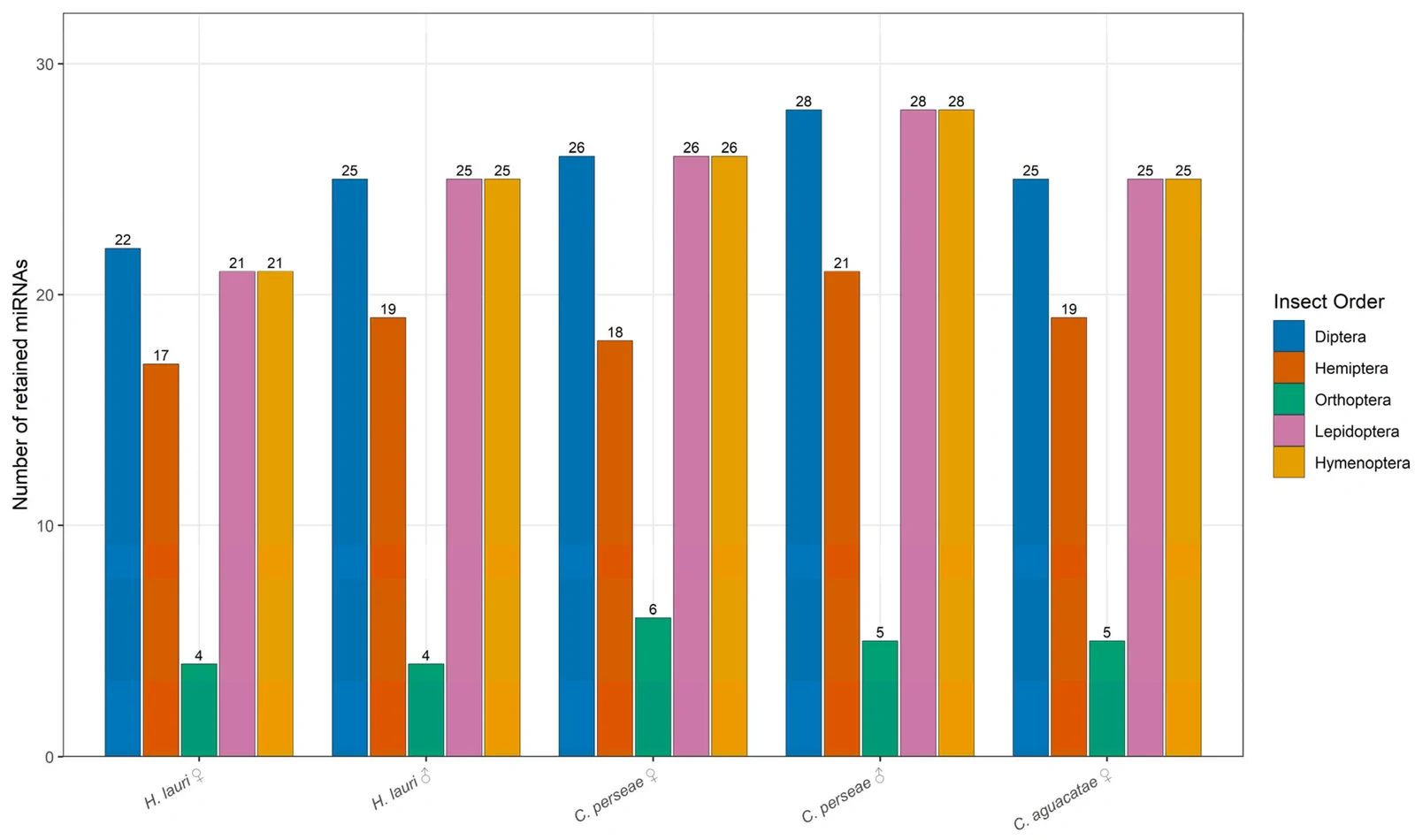
Cross-reference support for retained mature miRNAs across insect-order reference sets. Bars show the number of retained mature miRNA annotations supported in each sequencing library when the mature_other reference sequences corresponded to Diptera, Hemiptera, Orthoptera, Lepidoptera, or Hymenoptera. Five separate miRDeep2 runs were performed for each library, differing only in the insect order used as the mature_other reference. Values above the bars indicate the number of supported annotations. These comparisons represent cross-reference annotation support and should not be interpreted as independent biological replicates or as a formal phylogenetic conservation analysis. ♀, female; ♂, male.

## Data Availability

The original contributions presented in this study are included in the article/Supplementary Materials. Further inquiries can be directed to the corresponding author.

## Supplementary Materials

The following supporting information can be downloaded at https://zenodo.org/records/22836933.
